# Exercising ethnobiological resilience in turbulent times and places: in memoriam Sayed Hussain (1998–2023)

**DOI:** 10.1186/s13002-023-00596-2

**Published:** 2023-06-07

**Authors:** Wahid Hussain, Wasim Abbas, Andrea Pieroni

**Affiliations:** 1 Department of Botany, Govt. Post Graduate College Parachinar, Parachinar, 26300 Pakistan; 2 Plant Ecology and Conservation Lab, Department of Plant Sciences, Quaid-I-Azam University, Islamabad, Pakistan; 3 University of Pollenzo, Piazza Vittorio Emanuele II 9, 12042 Pollenzo, Italy; 4 Department of Medical Analysis, Tishk International University, Erbil, 44001 Kurdistan Iraq

**Keywords:** Ethnobiology, Conflict, Kurram, Minorities, Marginalization

## Abstract

On May 4, 2023, four schoolteachers and four drivers, including the young ethnobotanist Sayed Hussain, lost their lives at their school, massacred by religious extremists in the village of Teri Mangal, Kurram District, NW Pakistan, near the Pakistani-Afghan border. Ethnobiologists working in this area believe in the power of education and community-centered rural development as prominent tools for bringing about decent sustainable livelihoods in the near future and ultimately fostering social cohesion, tolerance, and peace. Ethnobiology was expressly conceived and designed to play a pivotal role in celebrating the richness of diversity of both indigenous and minority groups and especially to stop their oppression and discrimination, building the conditions for providing them true agency in their inalienable right to shape a decent future for their children. Field ethnobiologists in Kurram feel the palpable social tension, the fears local people confront daily and even sometimes the reluctance of a few community members to discuss and share their folk knowledge, while at other times, the burden of accessing militarily controlled areas and territories affected by landmines made their field research unfeasible. Nevertheless, ethnobiologists conducting field studies and navigating through these major difficulties exercise their daily resilience daily and believe in the power of the continuous dialogue between local knowledge holders and scholars.

The Journal of Ethnobiology and Ethnomedicine has never, until now, dedicated an editorial to an ethnobiologist who passed away, but we deliberately decided to make an exception on this occasion. On May 4, 2023, four schoolteachers and four drivers, including the young ethnobotanist Sayed Hussain (Fig. 1), lost their lives at their school, massacred by the religious extremists in the village of Teri Mangal, Kurram District, NW Pakistan, near the Pakistani-Afghan border. Sayed Hussain was a very engaged and dedicated young scholar, extremely sensitive and caring, who conducted fieldwork in Kurram [1] and was part of a group of courageous researchers—among them the two first authors of this editorial—who are working in this very critical area of the Hindukush. Sayed also wanted to be a teacher, while continuing his higher education, particularly in an area where education is needed the most. Therefore, a few months ago, he accepted a position in a high school in a radicalized area of upper Kurram, believing that the links he had built in the field over the years with these people could offer him a safe haven. The overall tragedy of this massacre of teachers garnered some attention in Western media, but it has left the authors of this editorial, his peers, and the whole ethnobiological family devastated and in indescribable pain.

**Fig. 1:**
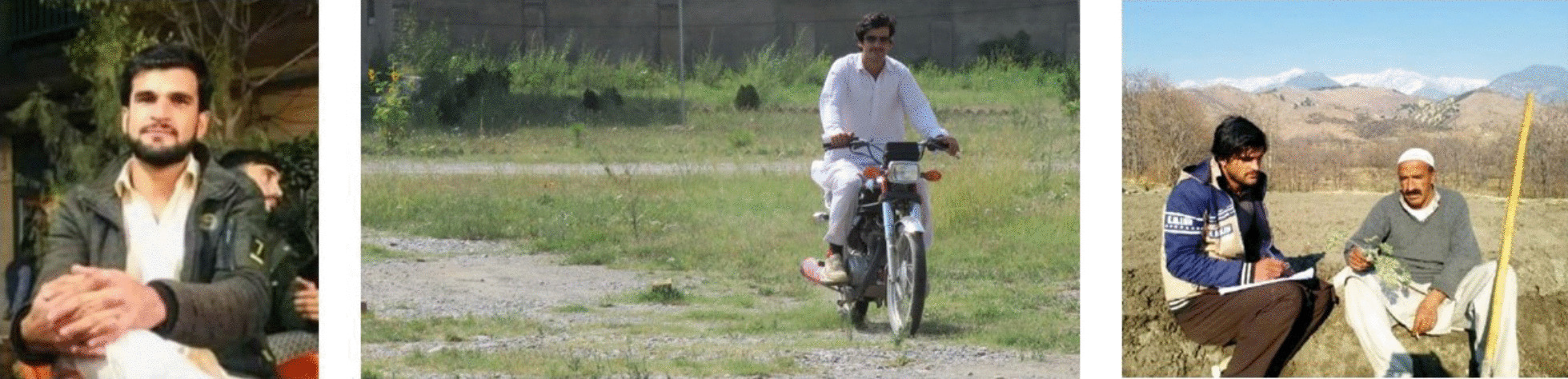
Sayed Hussain in his last picture before being massacred and during his ethnobiological fieldwork in Kurram

Over the past few decades, several local communities inhabiting the northwestern border of Pakistan have been badly affected by religious extremism and terrorism, which has inevitably destabilized their social equilibria and possibly their local knowledge system as well. The terrain of Kurram District is hilly and mountainous, and most of the villages in the region are cut off from towns, apart from occasional visits. The Koh-e-Safaid Range (Tura Bura) has always been a favorite location for terrorists and Afghan warlords including the Taliban, Al-Qaida, and various smugglers. The geography and multi-ethnic and multi-religious composition of Kurram Valley have made it a hotspot for terrorist activities, and peace in the region is intimately linked to sociopolitical changes in neighboring Afghanistan. The area faced the worst form of militancy for two decades, with approximately 6,000 deaths, that eventually led to a successful military operation, which has, however, left deep wounds and affected multiple facets of the local sociocultural fabric. However, it still takes a long time to install permanent peace in any region, which is why the monster of sectarianism and terrorism is still consuming people and their emotions.

As the majority of the population in Kurram belongs to lower social classes, local wild edible and medicinal plants are often the only option at hand when it comes to ensuring food security and health care within the domestic arena. Because of the sociopolitical instability in Kurram, as well as the chronic lack of modern health care facilities and declining per capita income, local communities have, in recent years, become even more dependent on local plant resources and traditional knowledge.

Ethnobiologists working in this area, like the two first authors of this writing, firmly believe in the power of education and community-centered rural development as prominent tools for bringing about sustainable livelihoods in the near future and ultimately fostering social cohesion, tolerance, and peace. Ethnobiology was expressly conceived and designed to play a pivotal role in celebrating the richness of diversity of both indigenous and minority groups and especially to stop their oppression and discrimination, building the conditions for providing them true agency in their inalienable right to shape a decent future for their children [2]. Additionally, ethnobiology was created to be present there, where it is most needed, and to raise the voice of threatened communities. As Ignacio Ellacuría (1930–1989), esteemed philosopher, theologian, and later rector at the Universidad Centroamericana in El Salvador, used to say: “university should be present intellectually where it is needed: to provide science for those without science; to provide skills for those without skills; to be a voice for those without voices; to give intellectual support for those who do not possess the academic qualifications to make their rights legitimate” [3].

The fieldwork that local ethnobiologists do in Kurram—as in other critical areas of the globe—is therefore more important than ever, as most researchers from the rest of the country and the world do not want to risk their lives and embark on studies in problematic areas. The intimate relationships between biodiversity and cultural diversity remind us that the ethnobiological ethos is profoundly linked to both the biological and social implications of constantly, dynamically changing local nature knowledge systems. This places ethnobiology in a privileged position for co-proposing solutions to very complex issues, sometimes within ongoing hazardous sociopolitical frames and environmental threats [4]. The question that arises, however, is whether ethnobiology is able to implement concrete solutions for the wellbeing of local people in the short-term, which are often needed, especially in areas devastated by war and major disruptive events. The answer seems inconclusive thus far, but we advocate strengthening the efficacy of ethnobiological research and networking worldwide, both by co-creating and sharing designs, methodologies, practices, and especially applications, and by training younger generations, always maintaining a high level of self-criticism and reflection.

The first two authors of this editorial, during their fieldwork in Kurram, felt the palpable social tension, the fears local people confront daily and even sometimes the reluctance of a few community members to discuss and share their folk knowledge. At other times, the burden of accessing militarily controlled areas and territories affected by landmines made the field research unfeasible. Nevertheless, ethnobiologists conducting field studies and navigating through these major difficulties in Kurram, fully self-funding their passion and mission, exercise their resilience daily and believe in the power of this continuous and sometimes fatiguing dialogue between local knowledge/practices and science and that between local knowledge holders and scholars.

We are convinced that this work is fundamental and necessary because it is the right thing to do *hic et nunc*, and we call worldwide colleagues and scholars to join us in this effort, now, more than ever, to decently honor the memory of our sweet friend and colleague Sayed Hussain.

## Data Availability

Not applicable.
